# Determination and disposition of *meta*-iodobenzylguanidine in plasma and heart of transporter-deficient mice by UPLC-MS/MS

**DOI:** 10.1016/j.jchromb.2023.123699

**Published:** 2023-04-07

**Authors:** Zahra Talebi, Yan Jin, Sharyn D. Baker, Daniel Addison, Alex Sparreboom

**Affiliations:** a Division of Pharmaceutics and Pharmacology, College of Pharmacy, The Ohio State University, Columbus, OH, USA; b Division of Cardiovascular Medicine, Department of Medicine, The Ohio State University, Columbus, OH, USA

**Keywords:** Mouse plasma and heart, Pharmacokinetics, UPLC-MS/MS, mIBG

## Abstract

A simple LC-MS/MS method for the quantitative determination of the norepinephrine analogue *meta*-iodobenzyl-guanidine (mIBG) was developed and validated for mouse plasma and tissues, including salivary gland and heart. The assay procedure involved a one-step solvent extraction of mIBG and the internal standard N-(4-fluorobenzyl)-guandine from plasma or tissue homogenates with acetonitrile. An Accucore aQ column was used to separate analytes using a gradient elution with a total run time of 3.5 min. Validation studies with quality control samples processed on consecutive days revealed values for intra-day and inter-day precision of < 11.3%, with values for accuracy ranging 96.8–111%. Linear responses were observed over the entire calibration curves range (up to 100 ng/mL), and the lower limit of quantification was 0.1 ng/mL, using sample volumes of 5 μL. The developed method was successfully applied to evaluate the plasma pharmacokinetics and tissue distribution of mIBG in wild-type mice and animals lacking the organic cation transporters OCT1, OCT2, OCT3, and/or MATE1 to further understand mechanisms contributing to drug distribution and elimination and causes of inter-individual pharmacokinetic variability.

## 1. Introduction

*Meta*-iodobenzylguanidine (mIBG) is a structural analog of norepinephrine that, when labeled with radioactive iodine, can be used for adrenal cortex imaging or treatment of neuroblastoma and other neuroendocrine tumors [1]. It is taken up into tumor cells by the norepinephrine transporter (NET, SLC6A2), which is highly expressed in neuroendocrine tumors [2]. However, according to whole-body scintigraphy, in addition to NET-mediated absorption into tumor lesions, mIBG also accumulates extensively in several normal tissues, such as heart and salivary glands [3,4]. The estimated pKa of mIBG is 11.27, indicating that this compound will exist in a cationic form under normal physiological conditions and that it requires a transport mechanism mediated by one or more members of the solute carrier class to enter cells. Consistent with this supposition, *in vitro* studies performed in heterologous expression models have confirmed that mIBG is a transported substrate for various organic cation transporters expressed in organs of relevance to the distribution and elimination of mIBG, including the liver (OCT1), the kidney (OCT2; MATE1; MATE2-K), heart (OCT3; MATE1) [5], and salivary glands (OCT3) [6–8].

In line with the known features of the mIBG tissue distribution profile, it has been suggested that concurrent administration of pharmacological inhibitors of organic cation transporters can restrict access of mIBG to healthy tissues, ameliorate injurious events of [^131^I]-mIBG, and improve its uptake in and radiotherapeutic effectiveness against tumors [9,10]. However, mechanistic details of the distribution and elimination pathways of mIBG and causal demonstration of specific organic cation transporters being involved in these processes remain lacking. Previously, Quiñones et al. reported an LC-MS/MS method for the measurement of mIBG in mouse plasma and liver, but the procedure required 20-μL sample volumes to meet the sensitivity requirement and involved a labor intensive, three-step deproteination extraction. Against this background, we set out to develop and validate a sensitive method for the determination of mIBG in mouse plasma and tissue homogenates based on liquid chromatography-tandem mass spectrometry that is adaptable to microvolumes of sample. The developed method was successfully applied to evaluate the pharmacokinetic profile of mIBG in plasma and tissues of mice carrying organic cation transporter deficiencies. This analysis resulted in the identification of OCT3 as an uptake transporter of mIBG in various tissues that can be targeted genetically and is sensitive to pharmacological inhibition.

## 2. Materials and methods

### 2.1. Chemical and reagents

Reference standards of mIBG hemisulfate and N-(4-fluorobenzyl)-guandine (nfBG), used as an internal standard for the analytical method, and dimethyl sulfoxide (DMSO) were purchased from Sigma-Aldrich (Burlington, MA, USA). All other reagents, including formic acid (FA), were obtained from Fisher Scientific (Fair Lawn, NJ, USA). Drug-free whole blood was obtained from untreated wild-type mice on an FVB background (Taconic Biosciences, Cambridge City, IN, USA), collected into heparin-containing tubes, centrifuged at 1,500 × *g* for 5 min, and the resulting supernatant was used as blank plasma.

### 2.2. Instrument and chromatographic conditions

All studies were performed using a Thermo Fisher Scientific Quantiva triple quadrupole mass spectrometer and a Vanquish UHPLC. For analyte separation, a C18 AQUASIL guard cartridge (2.1 mm × 10 mm, dp = 3 μm, Thermo Fisher Scientific) was used with an Accucore aQ column (2.1 mm × 100 mm, dp = 2.6 μm, Thermo Fisher Scientific). The autosampler and column were kept at 4 and 40 °C, respectively. A gradient elution was employed for 3.5 min at a flow rate of 0.45 mL/min using solvent A (0.1% FA in water) and solvent B (0.1% FA in acetonitrile-methanol, 50:50 v:v) as the mobile phase. Following were the gradient conditions: 0–0.5 min, 3% B; 0.5–2.0 min, 3% to 95% B; 2.0–3.0 min, 95% B; 3.0–3.01 min 95% to 3%; 3.01–3.5 min, 3% B. Aliquots of 3 μL of the extracted samples were injected. Contamination of the mass spectrometer was minimized by directing the flow using a diverter valve between 0.5 and 3.0 min into the mass spectrometer and redirecting it into waste otherwise.

The mass spectrometer’s settings for the sheath gas, aux gas, sweep gas, ion transfer tube, and vaporizer temperatures were 20 Arb, 12 Arb, 1.5 Arb, 300 °C, and 350 °C, respectively. The ion source was run in positive ion mode with the ion spray voltage set at 4000 V utilizing heated ESI. At a pressure of 1.5 mTorr, argon was used as the collision gas. For the analysis of mIBG and nfBG, the optimum selective reaction monitoring (SRM) transitions were used, and Table 1 lists the corresponding MS parameters for each. Thermo Scientific Xcalibur was used for data collection and processing (version 4.4.16.14, Thermo Fisher Scientific).

### 2.3. Preparation of stock and working solution of mIBG

Two individual primary stock solutions of mIBG and one stock solution of the internal standard were prepared in DMSO at final concentrations of 5 mg/mL. By using separate primary stock solutions of mIBG, working solutions were diluted in methanol for the further use of preparing calibration standards and QC samples at different concentrations. Methanolic working solutions of the internal standard were prepared from the stock at a concentration of 400 ng/mL. Solutions were stored at a temperature of −20 °C.

### 2.4. Calibration standards, QCs, and sample preparation

All calibration standards, QCs and sample were prepared by a single step deproteination method.

#### 2.4.1. Calibration standards and QCs preparation

The calibration curves were prepared by spiking 5 μL of respective blank biomatrix and 5 μL of 400 ng/mL internal standard in a 0.5 mL Eppendorf tube with 5 μL of appropriate mIBG working solution. Next, 85 μL of MeOH was added into the tube to create the calibration standards at 0.1, 0.5, 1, 5, 10, 250, 50 and 100 ng/mL. QC samples were prepared in batch by spiking required amount of mIBG working solution into either blank plasma or homogenized tissue. Next, 5 μL of QC sample at different concentrations was added into a 0.5 mL Eppendorf tube and mixed with 5 μL of 400 ng/mL internal standard and 90 μL of methanol to created QC samples at different concentrations, including the lower limit of quantification (LLOQ), low quality-control (LQC), medium quality-control (MQC), and high quality-control (HQC) at 0.1, 0.3, 40, 90 ng/mL, respectively. All tubes were then briefly vortexed and centrifuged for 9 min at 15,000 × *g* at 4 °C. Finally, the 60 μL of supernatant was transferred to the 96-well non-coated plastic microplates (Thermo Scientific) and a volume of 3 μL was injected into the system.

#### 2.4.2. Preparation of plasma samples

Frozen samples were thawed at room temperature prior to analysis. An aliquot of 5 μL of plasma was then placed into a 0.5 mL Eppendorf tube, following the same sample preparation protocol used for the extraction of QC samples (see section 2.4.1). Following that, 96-well non-coated plastic microplates (Thermo Scientific) were filled with 60 μL aliquots of the supernatant, and 3 μL was injected into the system.

#### 2.4.3. Preparation of salivary gland samples

To quantify mIBG in the salivary gland, the entire gland was weighted and then homogenized in a 3-fold volume (w/v) of distilled water at room temperature for 5 min using 0.9–2.0 mm stainless steel beads (Bullet Blender). An aliquot of 5-μL of homogenate tissue was transferred into a 0.5 mL Eppendorf tube, following the same sample preparation protocol used for the extraction of QC samples (see section 2.4.1). Next, 60-μL aliquots of the supernatant were transferred to the 96-well non-coated plastic microplate (Thermo Scientific), and 3 μL of well contents was injected into the system.

#### 2.4.4. Preparation of heart samples

Frozen hearts were thawed and homogenized in 3 volumes of distilled water at room temperature for 5 min, and 5-μL samples of the homogenates were transferred into a 0.5 mL Eppendorf tube, following the same sample preparation protocol used for the extraction of QC samples (see section 2.4.1). Next, 60-μL aliquots of the supernatant were transferred to the 96-well non-coated plastic microplate (Thermo Scientific), and 3 μL was injected into the system.

### 2.5. Method validation

#### 2.5.1. Linearity, accuracy, and precision

In order to generate the calibration curves, eight non-zero calibration standards (described in section in 2.4.1), a blank and a zero-calibration standard (blank spiked with internal standard) were ran daily in duplicate for four days and the peak area ratio of each analyte to their respective internal standard against the nominal concentrations of the compound was plotted daily. Weighted (1/x^2^) least-square regression was used to calculate the correlation coefficients where acceptable values needed to be higher than 0.99. Five replicates of the QC samples were analyzed at each level to assess intra-day accuracy. To determine inter-day accuracy and precision, five replicates of the described QCs were analyzed in four successive days, and accuracy was evaluated by calculating the percentage bias between the mean experimental value and the nominal concentration. The coefficient of variation (%CV) was calculated using standard equations to determine the intra- and inter-day precision. Values for accuracy were accepted if the percentage bias was within ± 15% of nominal values or ± 20% at the LLOQ, and values for precision were required to be < 15% or < 20% at the LLOQ. Statistical analyses were performed using validated Microsoft Excel calculation sheet and SPSS version 26 (SPSS, Inc., Chicago IL, USA).

#### 2.5.2. Specificity and selectivity

Specificity and selectivity were tested by analyzing six different lots of plasma and tissue samples from untreated mice to investigate potential chromatographic interference from endogenous components in plasma and/or tissues at the retention times of mIBG or the internal standard.

#### 2.5.3. Matrix effects and extraction recovery

Matrix effects that can be caused in biological samples by ionization competition between analytes of interest and the presence of matrix components were evaluated at the LQC, MQC and HQC samples by comparing the peak responses of mIBG and the internal standard observed in mobile phase solution and mouse plasma or homogenized tissue (salivary gland or hearts). In order to derive values for extraction recovery, the mean response observed in extracted samples was divided by the mean response in unextracted samples were evaluated at the respective LQC, MQC, and HQC samples, analyzed in triplicate. Recovery was assessed by calculating %CV, which for each analyte at different levels should always be < 15%.

#### 2.5.4. Carryover

To evaluate carryover, analyte concentration was assessed in a zero-calibration standard or an LLOQ sample, which was injected immediately after an ULOQ standard; six replicates were performed for each above-described assessment. If the analyte concentration in all zero calibration standards were below the limit of quantification and the percentage deviation from the LLOQ in at least 4 out of 6 samples was within 20%, the degree of carryover was considered negligible.

#### 2.5.5. Dilution integrity

To determine dilution integrity, 10x concentrated mouse plasma and tissues containing mIBG compared to respective HQCs, were tested followed by a 1:10 (v/v) dilution in the respective dissolving matrices to measure accuracy and precision, where acceptance criteria was set at an average percentage bias within 15.0 % of the nominal concentration and a %CV of < 15%, respectively.

#### 2.5.6. Stability

The stability of mIBG in mouse plasma and tissue homogenates (heart and salivary gland) was assessed by maintaining the concentrations at LQC and HQC under various storage conditions, including at room temperature for 6 h, in the autosampler for 24 h at 4 °C, 3 freeze–thaw cycles at −80 °C, and bench-top, autosampler and freeze–thaw stabilities.

### 2.6. In vivo pharmacokinetics studies

#### 2.6.1. Animal studies

*In vivo* experiments were performed in 10–18 weeks old male and female FVB mice from an inbred strain (weight, 20–30 g). Wild-type mice as well as our in-house genetically modified mice with a deficiency of OCT1 and OCT2, MATE1, or OCT3, and mice without OCT1, OCT2, and MATE1 were used in all experiments. The Ohio State University’s Animal Care and Use Committee, part of the University Laboratory Animal Resources (ULAR) organization, approved the experiments. Mice had free access to water and a normal meal while being housed in a temperature- and light-controlled facility. For *in vivo* studies, mIBG was prepared for intravenous administration by dissolving the powder in sterile phosphate-buffered saline (PBS) to create a 3 mg/mL solution so that the final dose in each mouse would be 15 mg/kg. Pharmacokinetic studies were performed as previously described [11]. Briefly, about 30 μL of whole blood samples collected from each mouse at 5 min, 15 min, 30 min, 1 h, 2 h, and 4 h after mIBG administration. The first three samples were obtained from the submandibular vein, the next two from the *retro*-orbital venous plexus, and the final sample by cardiac puncture. Blood samples were centrifuged at 1,500 × *g* (5 min) and plasma fractions were stored at −80 °C until analysis. All organs were collected with a surgical kit and rinsed in chilled PBS after collection and then were frozen on dry ice and stored at −80 °C.

#### 2.6.2. Pharmacokinetic data analysis

To determine pharmacokinetic parameters of mIBG, non-compartmental analyses were performed using Phoenix WinNonLin version 8.0 (Certara, Princeton, NJ, USA). The data from the log concentration vs. time curves were visually inspected to determine the peak plasma concentration (Cmax). To determine the area under the plasma concentration–time curve, the linear trapezoidal rule was applied (AUC). To compare the AUC of each treatment group and the amount of medication that accumulated in the tissues, normalized to the matching plasma concentration, a one-way analysis of variance (ANOVA) was carried out.

## 3. Results and discussion

### 3.1. Chromatographic and mass spectrometric conditions

**Fig. 1.**
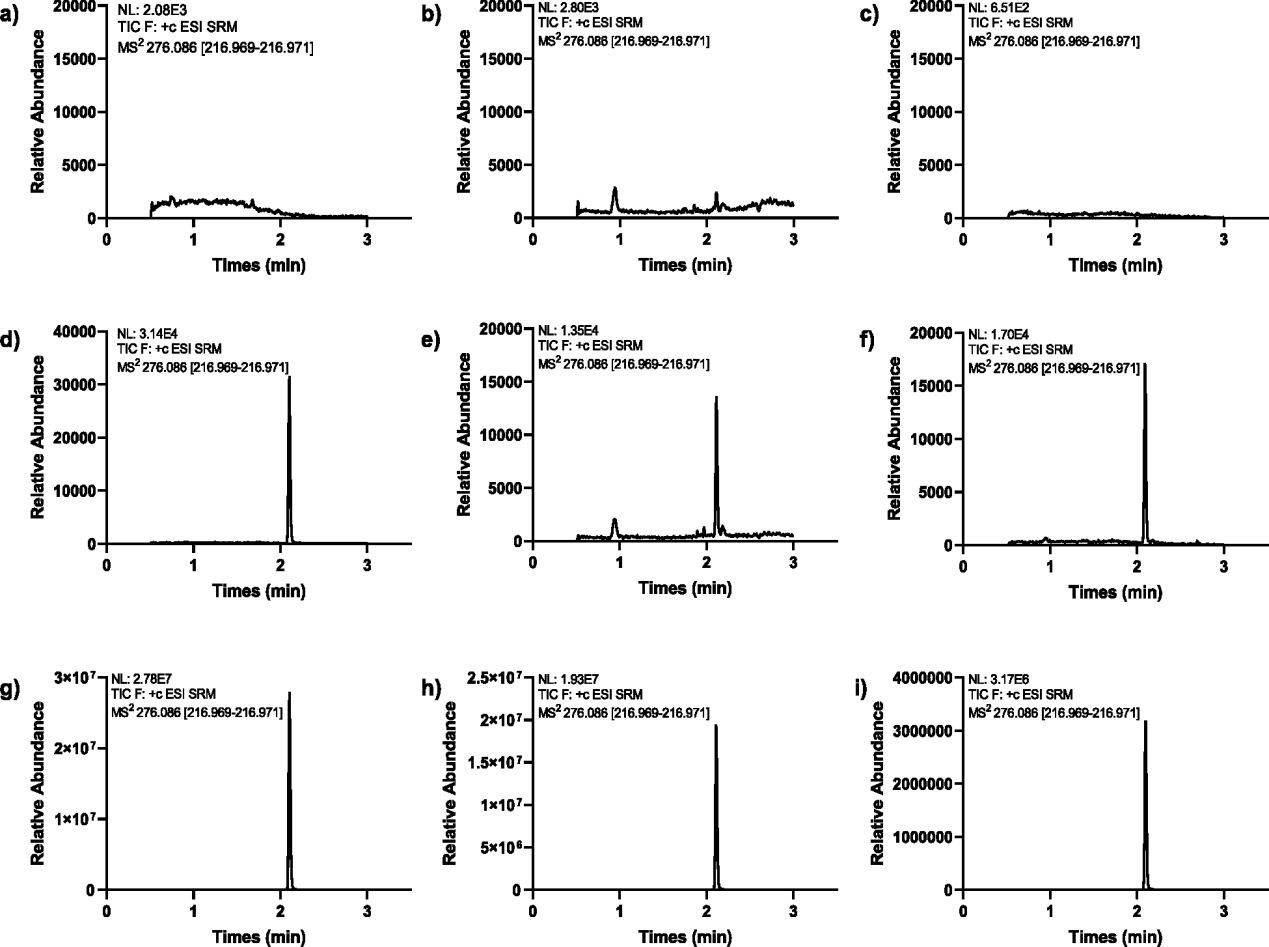
Representative chromatograms for the analysis of mIBG in mouse samples. Samples represent (a) untreated mouse plasma, (b) untreated salivary gland and (c) untreated heart of mouse, at the concentration 0.1 ng/mL (i.e. LLOQ) spiked into (d) untreated mouse plasma, (e) salivary gland and (f) heart of mouse, and in the treated samples collected from mice receiving an intravenous dose of mIBG at 15 mg/kg, including (g) mouse plasma, (h) salivary gland and (i) heart of mouse.

**Fig. 2.**
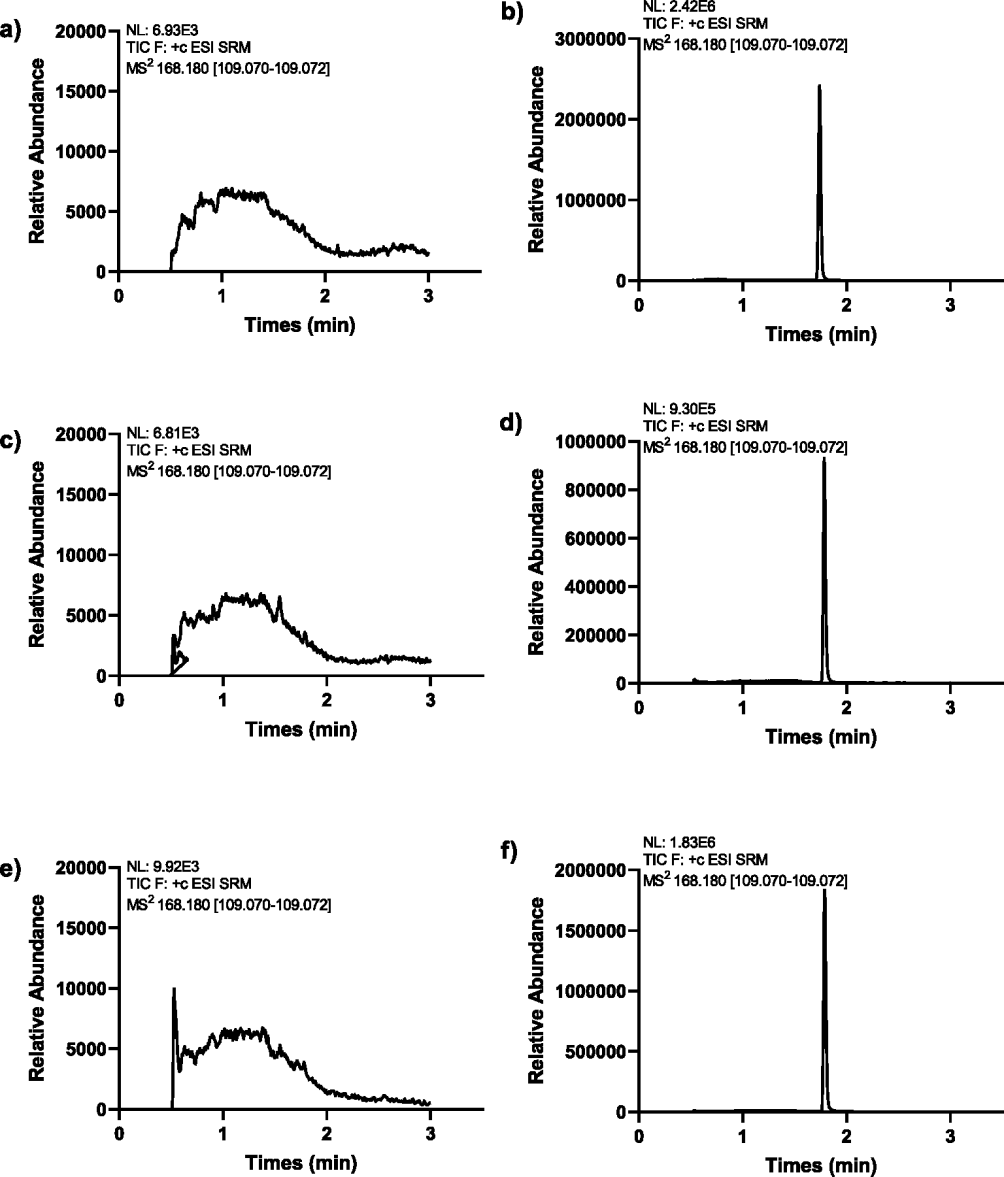
**Chromatograms of the internal standard**, niBG in (a) untreated and (b) 20 ng/mL-spiked in mouse plasma samples, (c) untreated and (d) 20 ng/mL-spiked in mouse salivary gland samples, (e) untreated and (f) 20 ng/mL-spiked in mouse heart samples.

MS setting of mIBG and the internal standard were obtained after performing various compound optimization steps in the mass spectrometer with an ESI source operating in positive ion mode. Detection was performed in the MRM mode at *m*/*z* 276.1 → 217.0 for mIBG and *m*/*z* 168.2 → 109.1 for the internal standard nFBG. Pertinent chromatographic conditions, including composition of the mobile phase, conditions of the elution gradient and flow rate, and type of stationary phase, pre-column, and column temperature, were optimized in several trials to improve resolution of the analytes, peak shape, and peak symmetry. Under the optimized conditions, the retention time averaged 2.10 min for mIBG (Fig. 1) and 1.78 min for nFBG (Fig. 2), using a total run time of 3.5 min.

### 3.2. Method validation

#### 3.2.1. Linearity, accuracy, and precision

The duplicated calibration curves of mIBG, generated on four consecutive days (n = 8) were found to be linear in a concentration range from 0.1 to 100 ng/mL. The average correlation coefficient observed during the validation procedures was > 0.99, indicating an acceptable degree of linearity within the tested concertation ranges of analytes in the relevant murine biomatrices.

Values for intra-day precision, inter-day precision, and accuracy for mIBG in mouse plasma and homogenates of salivary gland and heart are shown in Table 2. The observed percentage deviation from nominal values was always < 15% on each validation day at all concentrations, including the LLOQ. The values for intra-day precision and inter-day precision were also all<15%, suggesting that the present method has a satisfactory degree of accuracy and precision.

#### 3.2.2. Specificity and selectivity

Typical MRM chromatograms of plasma and tissues from untreated animals that were spiked with mIBG at concentrations representing the LLOQ, as well as samples from mIBG-treated and non-spiked samples from non-treated mice are shown in Fig. 1. No significant interfering peaks were observed around the retention time of mIBG and the internal standard (Fig. 2) during the analyses, supporting the specificity of the method.

#### 3.2.3. Matrix effect and extraction recovery

QC samples at concentration levels representing the LQC, MQC and HQC were assayed for matrix effects and evaluation of extraction recovery (Table 3). No significant matrix effects were observed in mouse plasma, heart, and salivary gland. Neat methanol was used as the reagent to study the extraction recovery of mIBG at LQC, MQC and HQC levels in mouse plasma and tissues, and the results are shown in Table 3. The overall extraction efficiency of mIBG when extracted from plasma, salivary gland and heart homogenates was higher than 80%, indicating that the chosen protein-precipitation step provides an effective sample pre-treatment procedure.

#### 3.2.4. Carryover

The degree of carryover was considered negligible for mIBG, as no representative peak in zero-calibrators, representing blank biomatrices spiked with only the internal standard, were observed. In addition, all of the LLOQ samples showed a percentage deviation from the nominal concentration that was within 7.30%, confirming minimal levels of carryover influencing the analysis.

#### 3.2.5. Dilution integrity

A dilution factor of 10x using mouse plasma and homogenized tissues as a diluent in a one-step process was established (Table 2). The parameters for precision observed for the 10-fold diluted QC samples prepared in plasma, salivary gland, and heart homogenates were 4.17%, 7.16%, and 8.97% (intra-day), 3.41%, 6.75% and 9.28% (inter-day), respectively. The observed percentage bias was between −3.20% and 10.5% in the respective matrices. These results indicate that biological samples containing concentrations higher than the ULQ can be diluted with respective blank matrices and re-analyzed so that concentrations are able to fall within the validated concentration range without measurable loss in accuracy or precision.

#### 3.2.6. Stability

The results from stability experiments with mIBG under various conditions are shown in Table 4, and indicated that mIBG present in the various matrices stored at ambient temperature remained stable for at least 6 h. In addition, no degradation of mIBG was observed in reconstituted samples kept at 4 °C in the autosampler for up to 24 h, or after three freeze–thaw cycles, suggesting that these conditions do not affect the integrity of mIBG in mouse plasma and tissue samples.

### 3.3. Pharmacokinetic studies

**Fig. 3.**
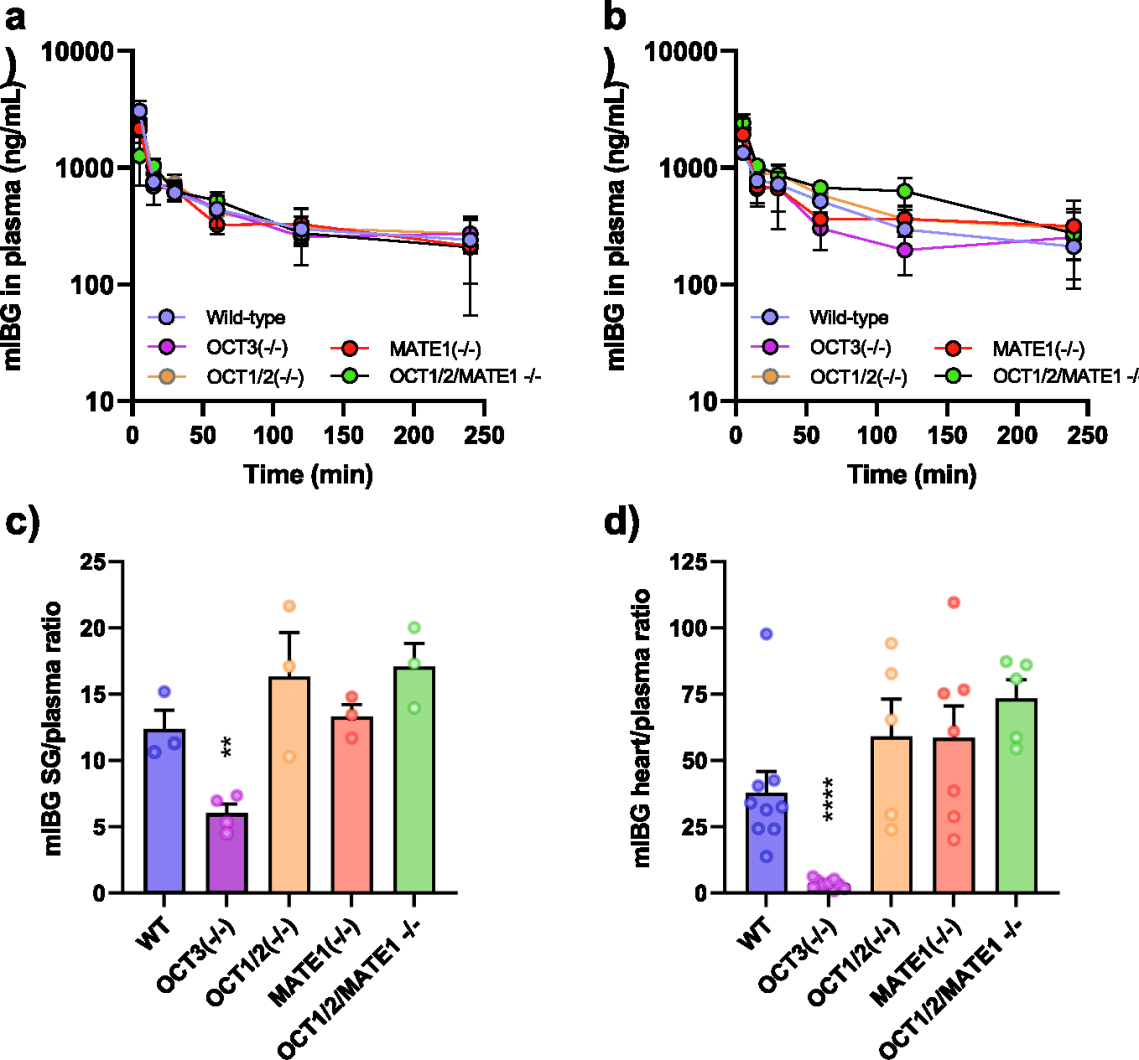
Pharmacokinetics of mIBG in mice with varying organic cation transporter genotypes. Plasma concentration–time profile of mIBG in male (a) and female mice (b) receiving a single intravenous dose of mIBG at 15 mg/kg. Levels of mIBG in the salivary gland (SG) (c) and heart (d) obtained 4 h after the administration of mIBG were normalized to plasma concentration. Five mice were dosed in each group (5 males and 5 females) except for salivary gland (5 males), and data represent mean values (symbols) and SD (error bars). In the heart/plasma ratios, data from males and females were combined. Group effects were evaluated by a one-way ANOVA, and statistical differences are indicated by **P < 0.01 and ****P < 0.0001.

The developed analytical method was next applied to measure plasma and tissue concentrations of mIBG in male and female mice with different organic cation transporter genotypes receiving the agent by intravenous bolus injection. The overall observed mean plasma concentration–time curves of mIBG in male mice (Fig. 3a) and female mice (Fig. 3b) receiving a dose of 15 mg/kg showed no statistically significant sexual dimorphism over the collected time interval (P > 0.05). While the observed AUC of mIBG in plasma was similar between wild-type mice and transporter-deficient animals, the levels of mIBG in murine salivary glands (Fig. 3c) and hearts (Fig. 3d) of OCT3-deficient mice were statistically significantly reduced compared to wild-type mice, consistent with a contribution of OCT3 to the uptake of mIBG in those tissues. Summarized pharmacokinetic parameters calculated using non-compartmental analysis are shown in Table 5. These findings are consistent with predictions that have been made for mIBG [1] and with published reports indicating that the levels of certain cationic substrates, such as tetraethylammonium, dehydrocorydaline, metformin, and doxorubicin, in the salivary gland and/or heart are impaired under OCT3-deficient conditions [5,12–15]. In contrast to observations made in OCT3-deficient mice, levels of mIBG in the salivary glands and hearts of mice deficient in OCT1, OCT2, and/or MATE1 were either unchanged or slightly increased compared to results obtained in wild-type mice (Fig. 3). It is worth noting that the observations made in MATE1-deficient mice are in agreement with recent observations for the MATE1 substrate drug dofetilide, which indicate that MATE1 acts as a cardiac efflux transporter and that its deficiency results in increased retention of certain substrates in the murine heart [16].

## 4. Conclusion

In this study, we developed and validated a robust and accurate UHPLC-MS/MS method for the quantitative determination of mIBG in mouse plasma and tissues, including salivary gland and heart. Compared to a previously reported assay to quantify concentrations of mIBG in mouse plasma and tissues [17], our method requires smaller sample volumes, effectively separates analytes in a shorter run time, and meet the demands of preclinical pharmacokinetic studies that involve serial sampling from the same animal. Validation results indicated that the developed method is sufficiently accurate, precise, and robust for measuring mIBG in samples collected from different murine matrices and is easily adaptable to routine analyses involving large numbers of samples. Implementation of the developed method in murine pharmacokinetic studies has revealed new insights into the role of organic cation transporters into the tissue distribution of mIBG *in vivo* and suggest a role of OCT3 in the uptake of mIBG in the heart and salivary gland. We are currently implementing this analytical method to further examine the influence of organic cation transporter inhibitors on the biodistribution of mIBG and to explore the utility of mIBG as a biomarker of cardiac OCT3 function.

## Figures and Tables

**Table 1:** MRM transitions and retention times for mIBG and the internal standard nFBG.

Analyte	RT (min)	Precursor (*m/z*)	Product (*m/z*)	Collision Energy (V)	RF Lens (V)

mIBG	2.10	276.1	217.0	23.24	64
nFBG	1.78	168.2	109.1	19.7	36

*Abbreviations:* RT, retention time.

**Table 2:** Accuracy and precision of mIBG analysis in mouse plasma and tissues.

Matrix Type	Level	Nominal Concentration (ng/ mL)	Inter-Day Precision (CV%, n = 5)	Intra-Day Precision (CV%, n = 20)	Accuracy (Bias%, n = 20)

Mouse Plasma	LLOQ	0.1	4.79	3.01	10.5
	LOQ	0.3	2.82	2.21	2.52
	MQC	40	3.16	2.58	3.86
	HQC	90	4.07	2.44	3.97
	Diluted-QC	900*	4.17	3.41	2.87
Mouse Salivary Gland	LLOQ	0.1	8.70	8.96	0.08
	LOQ	0.3	8.22	8.20	0.49
	MQC	40	7.77	7.67	1.08
	HQC	90	8.37	8.09	2.20
	Diluted-QC	900*	7.16	6.75	2.42
Mouse Heart	LLOQ	0.1	10.7	11.3	−3.20
	LOQ	0.3	8.93	7.87	−1.43
	MQC	40	9.27	8.62	−0.82
	HQC	90	9.17	8.72	0.49
	Diluted-QC	900*	8.97	9.28	−0.21

*Abbreviations:* LLOQ, lower limit of quantification; LQC, low quality-control; MQC, medium quality-control, and HQC, high quality-control, CV, coefficient of variation.

* QC samples were measured after 10x dilution using respective matrices.

**Table 3:** Matrix effect and recovery of mIBG in mouse plasma and tissues.

Matrix type	Conc. (ng/mL)	N	Matrix effect		Recovery	
			Mean matrix effect (%)	CV^a^ (%)	Mean recovery (%)	CV (%)

Plasma	0.3	3	93.2	3.95	104	3.22
	40	3	94.8	5.23	96.4	3.61
	90	3	92.5	7.89	108	1.88
Salivary gland	0.3	3	85.9	9.98	83.9	4.27
	40	3	87.8	8.17	83.2	6.67
	90	3	93.5	5.05	80.3	5.39
Heart	0.3	3	91.9	5.19	99.2	7.52
	40	3	90.2	5.76	87.9	2.37
	90	3	89.4	7.98	81.3	4.23

a Coefficient of variation.

**Table 4:** Stability of mIBG in mouse samples under various storage conditions.

	Conc. (ng/mL)	Sample Conditions					
		
		Bench-top stability^a^	Autosampler stability^b^	Freeze-thaw Stability^c^
		Mean deviation (%) of t = 0	CV^d^ (%)	Mean deviation (%) of t = 0	CV (%)	Mean deviation (%) of nominal Conc.	CV (%)

Plasma	0.3	110	8.60	103	9.93	104	8.03
	90	101	9.50	99.4	6.33	94.4	11.5
Salivary gland	0.3	98.7	4.32	97.6	11.1	94.7	7.42
	90	93.7	6.86	103	3.37	102	7.25
Heart	0.3	90.4	10.6	88.2	8.30	98.4	9.16
	90	102	10.7	97.8	8.55	92.5	8.20

a Stored at ambient temperature (25 °C) for 6 h.

b Stored the fresh samples at auto-sampler (4 °C) for 24 h.

c After three freeze–thaw cycles.

d coefficient of variation.

**Table 5:** Plasma Pharmacokinetic parameters of mIBG in wild-type and transporter deficient mice.*.

Genotype	Cmax (ng/mL)	AUC (ng × h/mL)

**Females**		
Wild-type	1350 ± 132.7	1720 ± 114.9
MATE1 ^−/−^	1920 ± 326.5	1840 ± 239.1
OCT1/2 ^−/−^	2000 ± 47.7	2180 ± 137.1
OCT3 ^−/−^	2170 ± 314.8	1600 ± 222.9
OCT1/2/MATE1 ^−/−^	2430 ± 168	2720 ± 106.5
**Males**		
Wild-type	3070 ± 188.9	2040 ± 134.3
MATE1 ^−/−^	2160 ± 193.7	1740 ± 178.3
OCT1/2 ^−/−^	3070 ± 396.4	2130 ± 99.3
OCT3 ^−/−^	2510 ± 433.2	1900 ± 97.6
OCT1/2/MATE1 ^−/−^	1260 ± 322.3	1680 ± 110.5

*Abbreviations:* Cmax, peak plasma concentration; AUC, area under the plasma concentration versus time profile.

* Results represent mean and SE of observations made in 6 animals per group each receiving a single intravenous dose of mIBG at 15 mg/kg.

## Data Availability

Data will be made available on request.

## Abbreviations:

mIBG: *meta*-iodobenzylguanidine
